# Estrogen via GPER downregulated HIF-1a and MIF expression, attenuated cardiac arrhythmias, and myocardial inflammation during hypobaric hypoxia

**DOI:** 10.1186/s10020-025-01144-2

**Published:** 2025-03-20

**Authors:** Prosperl Ivette Wowui, Richard Mprah, Marie Louise Ndzie Noah, Joseph Adu-Amankwaah, Anastasia Wemaaatu Lamawura Kanoseh, Li Tao, Diana Chulu, Simon Kumah Yalley, Saffia Shaheen, Hong Sun

**Affiliations:** 1 Department of Physiology, School of Basic Medical Sciences, Xuzhou Medical University, 209 Tongshan Road, Xuzhou, 221004 Jiangsu China; 2 Xuzhou Key Laboratory of Physiological Function and Injury, Xuzhou Medical University, Xuzhou, China; 3 National Demonstration Center for Experimental Basic Medical Science Education, Xuzhou Medical University, Xuzhou, China

**Keywords:** Hypobaric hypoxia, Estrogen, Macrophage migration inhibitory factor, Hypoxia-inducible factor-1α, G-protein-coupled estrogen receptor, Myocardial inflammation

## Abstract

**Background:**

The human body is highly dependent on adequate oxygenation of the cellular space for physiologic homeostasis mediation. The insufficient oxygenation of the cellular space leads to hypoxia. Hypobaric hypoxia (HH) is the reduction in oxygen partial pressure and atmospheric pressure during ascent to high altitudes. This state induces a maladaptive response. Women and how hormones like estrogen influence hypoxia have not been explored with most research being conducted on males. In this study, we investigated the effects of estrogen and GPER on HIF-1a and MIF expression, cardiac arrhythmias, and inflammation during hypobaric hypoxia.

**Methods:**

Ovariectomy and SHAM operations were done on FVB wild-type (WT) female mice. 2 weeks after the operation, the mice were treated with estrogen (40 mg/kg) as a therapeutic intervention and placed in a hypoxic chamber at an altitude of 6000 m for 7 days. Cardiac electrical activity was assessed using electrocardiography. Alterations in protein expression, inflammatory, and GPER pathways were investigated using western blotting, ELISA, and immunofluorescence. Histological assessment was performed using Masson’s trichrome staining. Peritoneal macrophages were isolated for in vitro study.

**Results:**

Under hypobaric hypoxia (HH), the ovariectomized (OVX) group showed increased macrophage migration inhibitory factor (MIF) and hypoxia-inducible factor-1 alpha (HIF-1α) expression. In contrast, these factors were downregulated in the estrogen-treated and control groups. HH also caused cardiac inflammation and fibrosis, especially in the OVX + HH group, which had elevated proinflammatory cytokines (IL-1β, IL-6, TNF-α) and decreased anti-inflammatory cytokines (TGF-β, IL-10). Inhibition with G15 (a GPER antagonist) increased MIF and HIF-1α, whereas activation with G1 (a GPER agonist) decreased their expression, highlighting GPER’s crucial role in regulating MIF during HH.

**Conclusion:**

Estrogen regulates HIF-1α and MIF expression through the GPER during hypobaric hypoxia, suggesting a potential therapeutic pathway to mitigate maladaptive responses during high-altitude ascent.

**Graphical Abstract:**

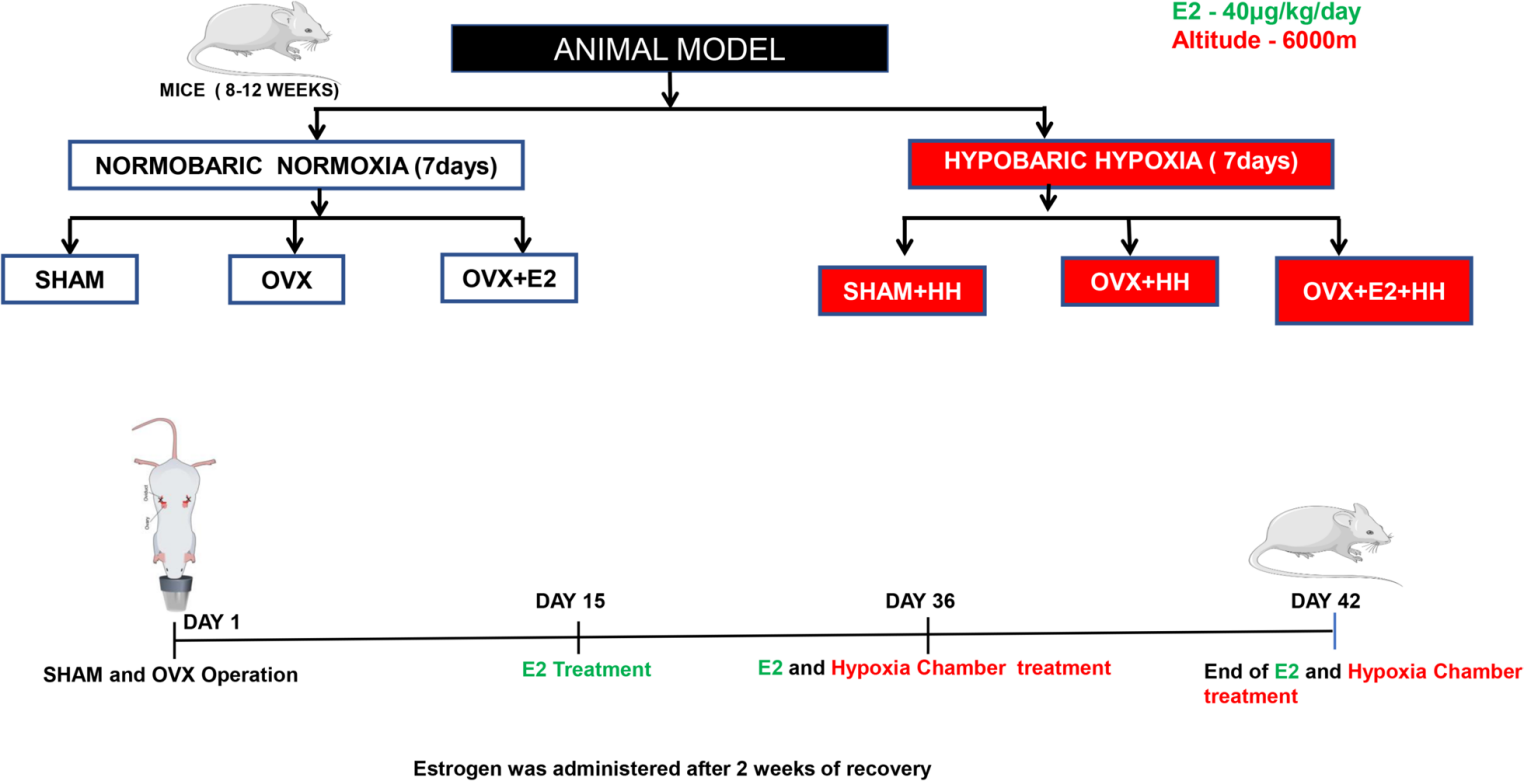

## Introduction

The human body relies on adequate oxygenation of cells to maintain physiological homeostasis. When the oxygen supply is insufficient, hypoxia occurs. It is estimated that approximately 81.6 million people live at altitudes above 2500 m, with some being permanent residents and others engaging in recreational activities. (Tremblay and Ainslie 2021) As ascent to higher altitudes increases, individuals experience hypobaric hypoxia (HH) due to the reduced partial pressure of oxygen and atmospheric pressure. This leads to decreased tissue oxygenation and various metabolic and physiological changes. (Eftedal et al. 2023) People exposed to HH commonly experience acute mountain sickness, hyperventilation, and elevated heart rate. (West 2012).

In response to HH, the body adapts by increasing red blood cell production, lung capacity, and angiogenesis (D'Alessandro et al. 2016). However, prolonged exposure to high altitudes can result in maladaptive responses, including inflammation, metabolic shifts, and adverse cardiovascular outcomes. (Bigham and Lee 2014) These effects are typically more severe in males and postmenopausal women, suggesting that female physiology might offer protective advantages during high-altitude exposure. (Joseph et al. 2000).

Estrogen (17β estradiol), a key female steroid hormone, is known to exert cardioprotective effects through its receptors: estrogen receptor alpha (ERα), estrogen receptor beta (ERβ), and the G-protein-coupled estrogen receptor (GPER) (Prossnitz and Barton 2011; Aryan et al. 2020). Estrogen has demonstrated cardioprotective effects, particularly in attenuating myocardial injury-reperfusion (MI/R) injury and inhibiting endoplasmic reticulum (ER) stress. (Chen et al. 2022) ERα and ERβ are nuclear receptors that regulate gene transcription following translocation into the nucleus under stress conditions (Yaşar et al. 2017). These nuclear-mediated effects are generally slower than the rapid signaling observed with GPER.

GPER, unlike classical estrogen receptors, is expressed both on the plasma membrane and intracellularly, enabling a rapid signaling response. GPER mediates its effects via the PI3K/AKT pathway, influencing various cardiac cell types such as cardiomyocytes, fibroblasts, and endothelial cells (Xu et al. 2023). This receptor regulates heart function, cardiac hypertrophy, and calcium channels (Groban et al. 2020). Studies have shown that during hypoxia-induced pulmonary hypertension, ERβ expression increases, attenuating pulmonary vascular remodeling and reducing HIF-1α expression. (Frump et al. 2018) However, the role of GPER in HH remains underexplored and warrants further investigation.

Macrophage migration inhibitory factor (MIF) is a pleiotropic cytokine traditionally associated with immune responses. Initially discovered in immune cells, MIF has since been found in cardiomyocytes and endothelial cells, where it regulates inflammation. (Calandra and Roger 2003) MIF has been implicated in several disease conditions, including hypertension, tumors, and acute myocardial dysfunction. (Jalce and Guignabert 2020) As a cytokine, MIF contributes to inflammation, impairing physiological function (Grieb et al. 2010). In high-altitude exposure, MIF expression is regulated by hypoxic conditions, with decreased MIF levels observed in women exposed to HH. (Verratti et al. 2017) In contrast, male rats exposed to HH show an upregulation of MIF expression in their lungs, indicating a possible gender difference in MIF regulation during HH. (Zhang et al. 2012) Consistently, estrogen has been reported to modulate MIF expression. For example, estrogen's ability to attenuate MIF expression has been observed in cutaneous wound healing. (Ashcroft et al. 2003).

Hypoxia-inducible factor (HIF) is a key regulator of cellular responses to oxygen availability, with HIF-1α playing a pivotal role in mediating adaptations to low-oxygen conditions. (Carroll and Ashcroft 2006; Cinque et al. 2022) Under normal oxygen levels, HIF-1α is degraded through hydroxylation and subsequent proteasomal degradation. (Berra et al. 2003; Iacobini et al. 2022) However, during hypoxia, HIF-1α accumulates, triggering transcriptional changes that help cells adapt to oxygen deprivation. (Adzigbli et al. 2022) HIF-1α also induces a metabolic shift from oxidative phosphorylation to glycolysis, reducing oxygen consumption at the expense of mitochondrial damage and increased reactive oxygen species (ROS) production, which can impair cell survival. (Taylor and Scholz 2022; Miska et al. 2019). In chronic hypoxia, HIF-1α expression is elevated, leading to sustained inflammatory responses via the induction of MIF, which further contributes to pathological effects such as myocardial dysfunction. (Mirtschink et al. 2019; Harjacek 2021).

With the rapid expression of MIF, its induction of inflammation, and the cardioprotective function of estrogen during stress conditions, this study aims to explore how estrogen, particularly through the GPER pathway, contributes to female adaptation during HH. It also seeks to fill the gap in understanding female-specific physiological responses and highlight potential therapeutic targets related to estrogen and GPER in combating the adverse effects of HH.

## Method and materials

### Experimental animal protocol

Eight to twelve-week-old wild-type FVB female mice were used in this study with mice in the same estrous cycle described in our previous work (Adu-Amankwaah et al. 2024). The mice were housed and fed in a hypoxia chamber (Guizhou Fenglei Aviation Machinery Co., Ltd., Guizhou, China: FLYDWC50-IIA). Hypobaric hypoxia (HH) was induced by increasing altitude from 3000 m for 10 min, then to 4500 m for 10 min, followed by 5500 m for 20 min before finally increasing to 6000 m altitude for 7 days. Mice in the control group were housed and fed in a normobaric normoxia (NN) environment (with ambient oxygen percentage) for 7 days.

### Ovariectomy

FVB wild-type female mice were sham-operated and ovariectomized, then divided into 6 groups comprising 6–8 mice each. Sham operation and ovariectomy were performed as follows. Female mice were anesthetized according to their body weight, and mice were laid on their abdomen. The exposed skin was prepared for an aseptic surgery with povidone-iodine, and a dorsal midline incision was made. A blunt dissection of the posterior abdominal muscle wall was performed with an incision done on each side of the spine to identify the ovaries. This was followed by ligating the proximal end of the uterine horn, where each ovary was excised, followed by the closure of the incision. The posterior abdominal muscle layers were sutured independently with 3-0vicryl sutures, and the skin layers were closed with a continuous silk suture. In the Sham group, the ovaries were exposed by incisions into the abdominal cavity, followed by abdominal muscle layers suturing. After two weeks post-surgery, mice were observed for wound healing and divided into 6 groups. 1. The Sham group was housed under both normobaric normoxia conditions (NN) and hypobaric hypoxia (HH) conditions, 2. OVX group, 3. OVX + E2 group, E2 (40 mg/kg/day) (Sigma, 50-28-2) was administered for 6 weeks, and mice were placed under the NN and HH condition for 7 days.

### Cell culture

Macrophages were isolated from the peritoneal cavity of female mice. Isolation was done as detailed previously (Ray and Dittel 2010). Briefly, mice were euthanized, dipped in 70% ethanol, and mounted on a Styrofoam block. The outer skin of the peritoneum was cut and gently pulled to expose the peritoneal cavity. 5 ml of ice-cold PBS containing 3% FBS was injected into the peritoneal cavity. Using a syringe, fluid is collected and spun in a centrifuge at 1500 RPM, and the cell is cultured in DMEM medium supplemented with (v/v) 10% heat-inactivated FBS and 1% penicillin for 24 h at 37 °C in a humidified atmosphere of 5% CO_2._ Cells were further divided into groups with diverse treatments administered and cultured for 24 h. Cells were treated with 1 nM of E2, 400 μM CoCL_2_ for the induction of hypoxia, 11 nM of GPER-1 agonist (G1), 20 nM of GPER-1 antagonist (G15) (Cayman; 14,673), 25 μM PI3K inhibitor (LY294002).

### Electrocardiography

Electrocardiography (ECG) data were performed with the 3-lead monopolar needle electrode from PowerLab systems (ADInstruments, North America), as previously described in (Doggett et al. 2018).

### Ventricular hypertrophy assessment

Body weight was measured and mice were sacrificed and the hearts were harvested. The right ventricle (RV) and left ventricle (LV) were cut. RV and LV hypertrophy were calculated as previously described (Mprah et al. 2022).

### Immunohistochemistry (IHC), immunofluorescence staining (IF), biochemical staining

#### Wheat germ agglutinin (WGA) staining

Hearts were sectioned and cryopreserved. The heart was fixed with 4% formaldehyde for 15 min at room temperature (RT), washed three times with PBS, and primed with HBSS for 15 min. The myocardial sections were incubated with WGA staining (Thermo Fisher Scientific; Alexa Flour, W11261) in the dark for 10 min and washed three times with PBS. DAPI and antifade were used to counterstain. The imaging was done at X60 magnification, and ImageJ (1.52a version; National Institute of Health USA) was used to detect cardiomyocyte surface area.

#### Masson’s trichrome staining

The myocardial sections were trichrome stained according to the manufacturer's (Solarbio; G1340) protocol. Results were analyzed with microscopy at X40 magnification, and collagen volume fractions (CVF) were analyzed with ImageJ.

#### Immunohistochemical (IHC) staining

F4/80 (Santa Cruz Biotechnology; sc-377009; 1:500), CD86 (Abcam; ab53004; 1:1000), and CD206 (Proteintech; 60143–1-Ig; 1:1000) IHC staining was done as previously described (Adu-Amankwaah et al. 2023), with a few sections optimized. The staining used frozen sections; therefore, the step for antigen retrieval was skipped in the described experiment, and the myocardial sections were fixed with 4% formaldehyde for 15 min before staining. The infiltration of F4/80, CD86 + , and CD206 + macrophages was observed at a magnification of X40 and quantified with ImageJ.

#### Immunofluorescence staining

The cultured and treated macrophages were fixed with (4%) formalin (n ≥ 2 ∗ 10^6^ cells per treatment group). The permeation of the cells was accomplished using pre-chilled methanol–acetone (ratio 1:1), triton x (0.1%) for 15 min. PBS with 1% BSA was prepared to block binding sites of non-specific antibodies for 1 h. Macrophages were incubated with treatment and left overnight at a temperature of 4 °C with MIF primary antibody (Abcam; ab7207), then rinsed with PBS and probed with R-PE-conjugated secondary antibody (SA00008-2: Proteintech) for 1 h at room temperature. The cells were washed with PBS and incubated with DAPI (Beyotime; P0131) nuclei staining for 3 min, followed by assessing and imaging the ratio of MIF expression in the nucleus and cytoplasm. The imaged cells were analyzed using ImageJ (1.53a version; National Institute of Health, Bethesda, MD, United States).

### Enzyme-linked immunosorbent assay (ELISA)

Heart tissues were homogenized with an adequate (500 µl–1000 µl) of PBS on ice and centrifuged at 4 °C 4000 rpm for 5 m. The supernatants were discarded, and the sediments of homogenized tissues were resuspended in 1 ml of PBS twice, and centrifuged again at 4 °C 4000 rpm for 5 m. The supernatants were again discarded to get rid of all the blood. A lysis buffer mixture (cocktail) containing RIPA and PMSF, in the ratio of 100:1, was prepared. About 150–250 μl of the RIPA with PMSF was added to the homogenized samples and kept on ice. The samples were intermittently vortexed for 6 × at 5 min intervals. They were then balanced into a centrifuge and spun at 4 °C 14000 rpm for 10 min. The supernatants were collected into appropriately labelled Eppendorf tubes and kept on ice, and the sediments were discarded.

Myocardial lysates derived from the in vivo model were used to assess the concentrations of proinflammatory cytokines (TNFα, IL-1β, and IL-6), anti-inflammatory cytokine (IL-10, TGF-β), and cardiac injury markers (ANP, BNP, and cTnI). Cell culture media supernatants from in vitro models were also used in the cell to examine the same proinflammatory and anti-inflammatory cytokines. IL-6 (JL20268; Jianglai Biotechnology), IL-1β (JL18442; Jianglai Biotechnology), TNF-α (JL10484; Jianglai Biotechnology), IL-10 (JL20242; Jianglai Biotechnology), TGF-β (JL13959; Jianglai Biotechnology), ANP (EK12636; Sabbiotech.), BNP (EK12906; Sabbiotech.); Troponin I (cTnI) (EK1821; Sabbiotech.). ELISAs were performed in triplicates and as per the manufacturer's instructions.

### Western blot

Hearts were harvested and homogenized, and a cocktail of RIPA buffer, protease, and phosphatase inhibitor (ratio 100:1:1) was added to extract proteins. The protein concentrations were normalized and denatured. Samples were run on 12% sodium dodecyl sulfate (SDS)-polyacrylamide gel electrophoresis and transferred onto 0.45 μm PVDF membrane (Millipore Immobilon®-P; IPVH08100). The membranes were blocked with 1% BSA in TBST, and the membrane was incubated in the antibody of interest overnight at a temperature of 4 °C. The proteins of interest are as follows: anti-MIF (Abcam; ab7207; 1:1000), anti-HIF-1α (Proteintech; 20,960–1-AP; 1:1000), anti-GATA4 (Abcam; ab84593; 1:1000), AKT (4685S:1:1000, Cell Signaling Technology), pAKT(1:1000, Cell Signaling Technology), ERK1/2(1:1000, Cell Signaling Technology), pERK1/2(Proteintech; 28,733-1-AP; 1:1000), COL 1, COL3A1, GPER-1(1:500, Abcam), PI3K(Cell Signaling Technology; 4255S; 1:1000), pPI3K(1:1000, Cell Signaling Technology), NF-κB(1:1000, Abmart), pNF-κB(1:1000, Abmart, GAPDH (Proteintech; 10,494-1-AP; 1:1000) and HRP-conjugated Goat Anti-Rabbit IgG(H + L) (Proteintech; SA00001-2; 1:1000). The in vitro, antibody includes; anti-MIF (Abcam; ab7207; 1:1000), anti-HIF-1α (Proteintech; 20,960-1-AP; 1:1000), GPER-1(Abcam, 1:500), COL 1; COL 3, PI3K (Abclonal; A11177; 1:1000), pPI3K(Beyotime), anti-GATA4 (Abcam; ab84593; 1:1000). Membranes were visualized using enhanced chemiluminescence (Tanon, China).

### Statistical analysis

The results of this study are presented as mean ± SEM. Analysis was done using GraphPad Prism (Software version 9.0.0). One-way ANOVA was utilized to compare three or more groups and two-way ANOVA was used to analyze grouped data. P-values < 0.05 relate to statistical significance.

## Results

### Estrogen mitigates abnormal cardiac electrical activity during hypobaric hypoxia

**Fig. 1:**
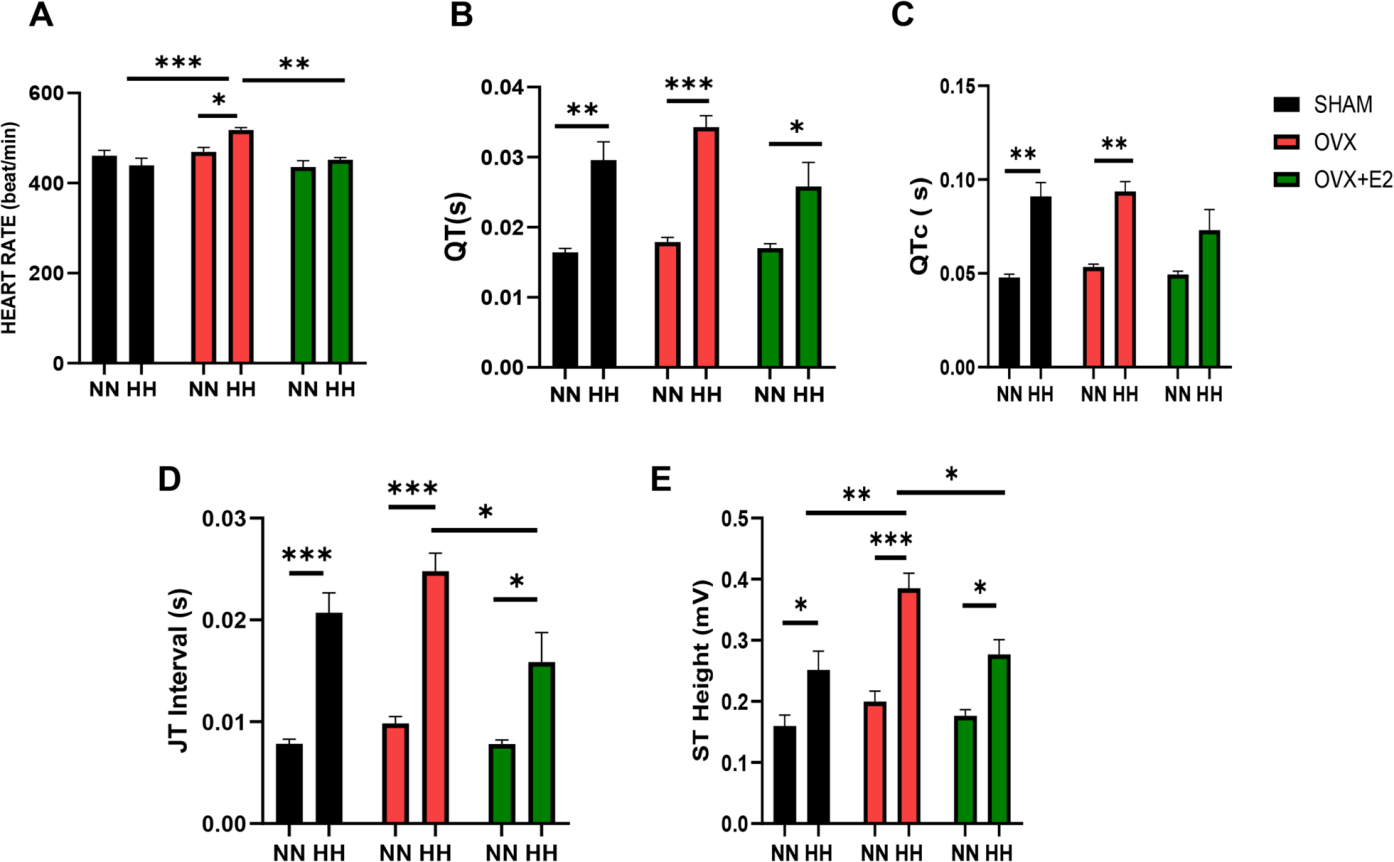
Effect of HH on cardiac activity **A** Graphical representation of Heart rates; **B** and **C**: Graphical representation of QT interval, QTc and **D** and **E:** representation of JT interval and ST height. Results are presented as mean$\pm \text{SEM}$. (n = 12–15), (*p < 0.05, **p < 0.01, ***p < 0.001), (TWO WAY ANOVA)

Cardiac function assessed at the end of animal models demonstrates that compared with the OVX + HH mice, the SHAM + HH group had a decreased heart rate (HR) (Fig. 1A) and prolonged QT interval and QTc (Fig. 1B and C). Furthermore, increased heart rate, JT interval and ST height were observed in the estrogen deficiency mice group (OVX + HH) compared to the estrogen-treated group (OVX + E2 + HH) and the Sham group (SHAM + HH), which showed improved cardiac electric activity (Fig. 1D & E). Similarly, control mice at normoxia (NN) had better cardiac electrical activity than those in hypobaric hypoxic (HH) conditions.

### Estrogen attenuated HH-induced cardiac morphological alteration and injury

**Fig. 2:**
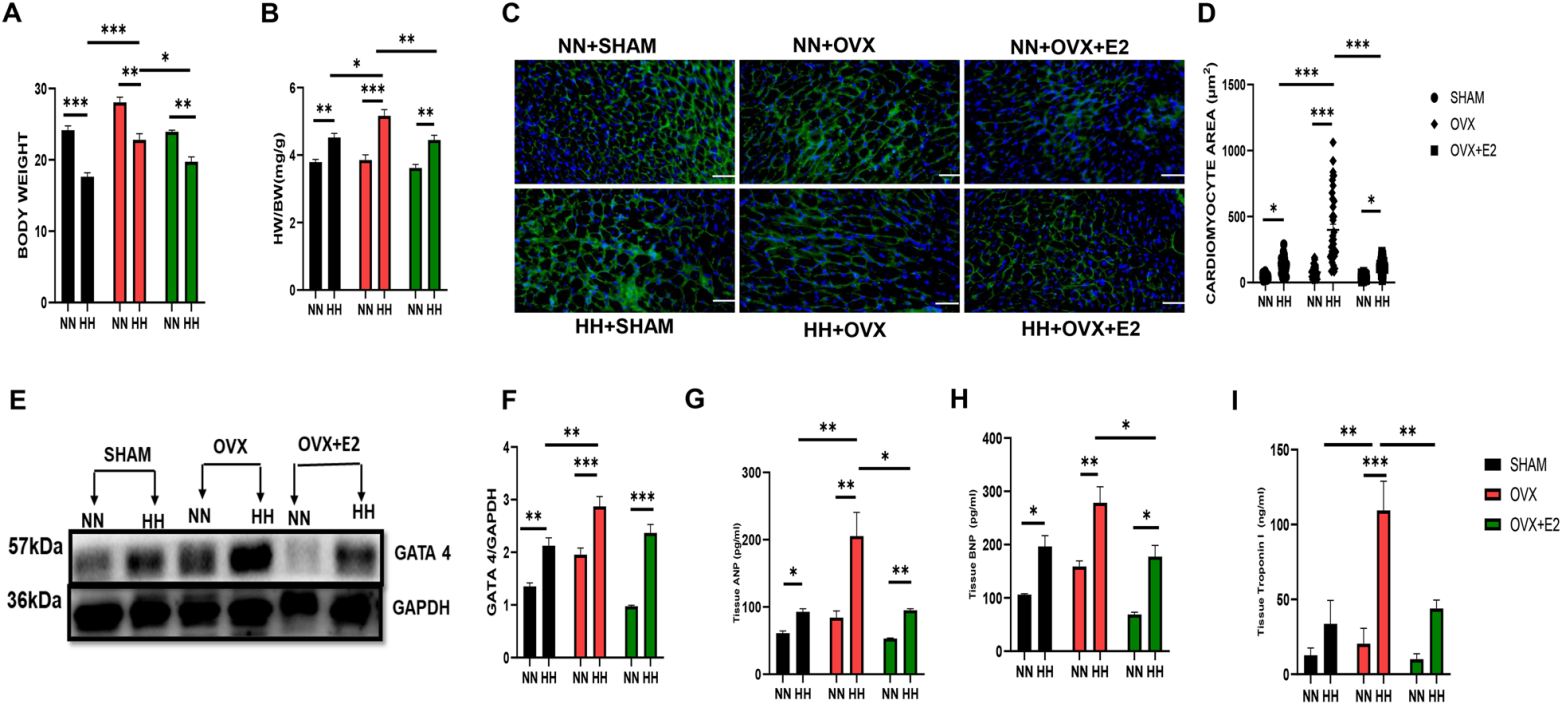
Effect of E2 on cardiac morphometric and injury markers. **A** & **B** Graphical representation of Body weight, and HW/BW. **C** & **D** Cardiomyocyte area. **E** & **F** Western blot band and graphical representation of blot **G**, **H**, **I**: Graphical representation of markers of hypertrophy and injury. This result is represented as a mean $\pm \text{SEM}.$ (n = 6) (*p < 0.05, **p < 0.01, ***p < 0.001), *ANP* Atrial Natriuretic Peptide, *BNP* Brain Natriuretic Peptide, *BW* Body weight, *cTnI* Cardiac troponin I, *ELISA* Enzyme-linked immunosorbent assay, *GATA4* GATA Binding Protein 4, *HW* Heart weight

**Fig. 3:**
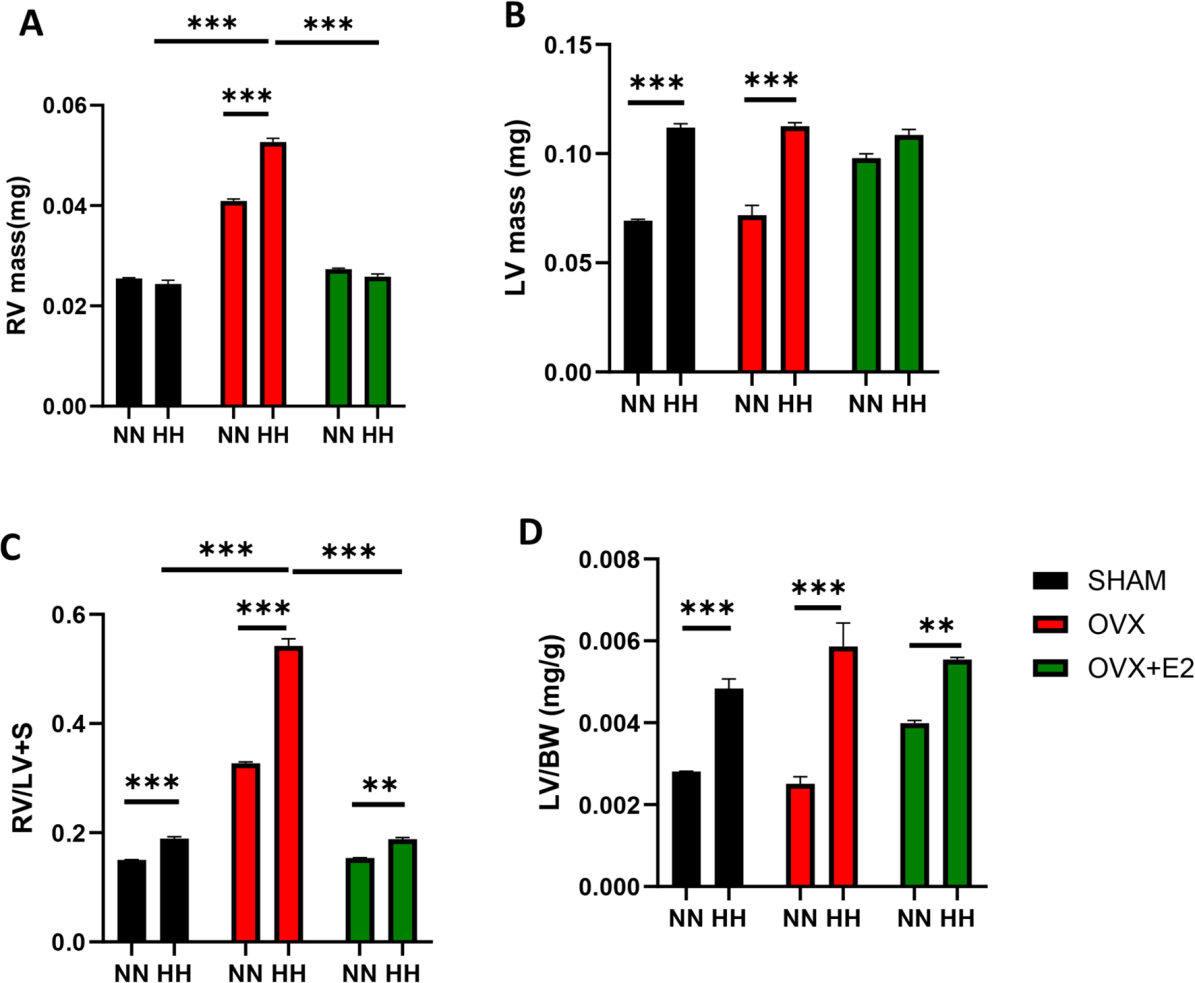
Effect of E2 on ventricular hypertrophy during HH. **A:** Graphical representation of RV mass. **B** LV mass. **C** RV/LV&S **D** LV/BW. This result is represented as a mean ± SEM. (n = 6) (*p < 0.05, **p < 0.01, ***p < 0.001), RV: Right ventricular, LV: Left ventricular, S: Septum

HH exposure resulted in significant cardiac hypertrophy with decreased body weight across all experimental groups compared to the normoxia controls (NN). However, between the HH groups, the ovariectomized (OVX + HH) group exhibited increased weight gain compared to the estrogen-treated groups. (Fig. 2A) correlating with the heart weight-to-body weight ratio (HW/BW) exhibiting a marked elevation in the OVX + HH cohort relative to the OVX + E2 + HH and SHAM + HH groups (Fig. 2B). Corroborating these observations, the analysis of the cardiomyocyte cross-sectional area exhibited significantly enlarged cardiomyocytes in the OVX + HH group relative to the SHAM + HH and OVX + E2 + HH groups (Fig. 2C&D) depicting possible structural changes. Consistent with these findings, the hypertrophic marker GATA4, a mediator of hypertrophy-related genes such as BNP and ANP, exhibited elevated expression levels in the estrogen-deficient OVX + HH cohort under HH conditions (Fig. 2E and F). The assessment of hypertrophy-related genes revealed augmented levels of atrial natriuretic peptide (ANP), brain natriuretic peptide (BNP), and cardiac troponin I (cTnI) in the OVX + HH group compared to the SHAM + HH and OVX + E2 + HH cohorts, where a significant reduction was observed (Fig. 2G, H and I). Hypobaric hypoxia is associated with ventricular hypertrophy. We analyzed the right and left ventricular mass and observed a significant increase in the HH group compared to NN (Fig. 3A and B). The ovariectomized group in the presence of hypoxia exhibited a significant increase in RV mass, and this was ameliorated in the estrogen-treated groups (Fig. 3A). Also, there was no significant increase in the LV mass between the ovariectomized and estrogen groups during hypoxia (Fig. 3B). To further confirm the hypertrophic state, we detected the RV/LV + Septum and the LV/BW and observed an increase in the right ventricular hypertrophy compared to the left ventricle during HH (Fig. 3C,D). This was observed with an increase in RV/LV + S in the OVX + HH group compared to the estrogen cohorts (Fig. 3C). Also, there was no significant increase in the LV/BW between the ovariectomized group and the estrogen-treated group during HH (Fig. 3D). Collectively, these results indicate that during hypobaric hypoxia exposure, estrogen deficiency, as observed in the OVX + HH group, significantly impacts cardiac morphology by inducing hypertrophic remodeling. This phenomenon was attenuated by endogenous (SHAM + HH) or exogenous (OVX + E2 + HH) estrogen, highlighting estrogen’s role in mitigating hypertrophy and improving cardiac structure.

### Estrogen ameliorated HH-induced cardiac inflammation and fibrosis

**Fig. 4:**
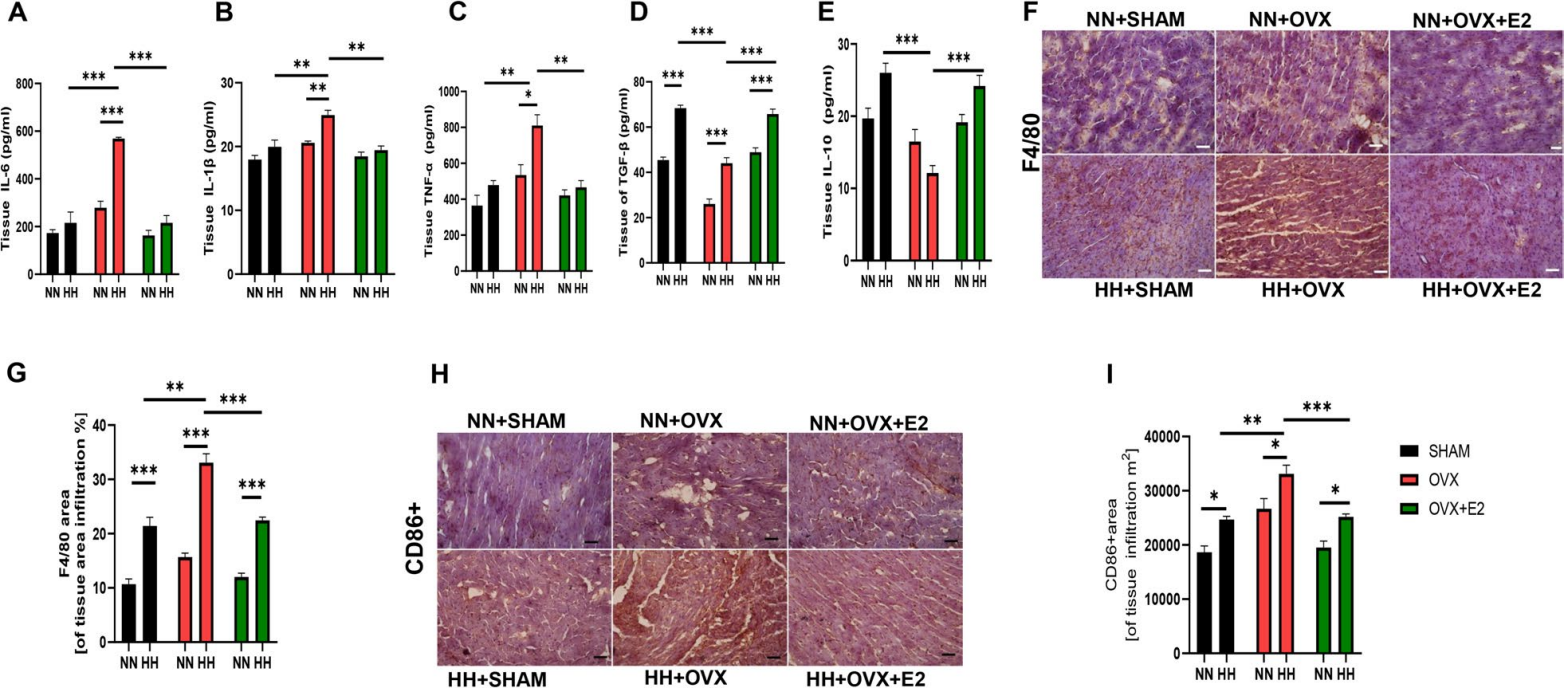
Effect of E2 on inflammation and macrophage infiltration during HH. **A-E:** Inflammatory cytokines; Interleukin IL-6, (IL)-1β, Tumor necrosis factor-α (TNF-α), IL-10, and transforming growth factor (TGF)-β concentrations assessed by ELISA using myocardia lysates. **F-I** Immunohistochemistry graphical presentations of F4/80 and CD86+ macrophages Results are presented as mean ± SEM. (*p < 0.05, **p < 0.01, ***p < 0.001),

Inflammation has been implicated in maladaptive responses during hypobaric hypoxia (Pham and Parikh 2021). Our assessment of the inflammatory state in cardiac tissue revealed an augmented inflammatory response in the estrogen-deficient ovariectomized (OVX + HH) group subjected to HH conditions. This was evidenced by the elevated expression of proinflammatory cytokines, including interleukin-6 (IL-6), IL-1β, and tumor necrosis factor-α (TNF-α), in the OVX + HH cohort (Fig. 4A–C). Conversely, the sham-operated (SHAM + HH) and estrogen-treated OVX (OVX + E2 + HH) groups exhibited suppressed inflammatory responses, accompanied by an increase in the anti-inflammatory cytokines transforming growth factor-β (TGF-β) and IL-10 (Fig. 4D and E).

**Fig. 5:**
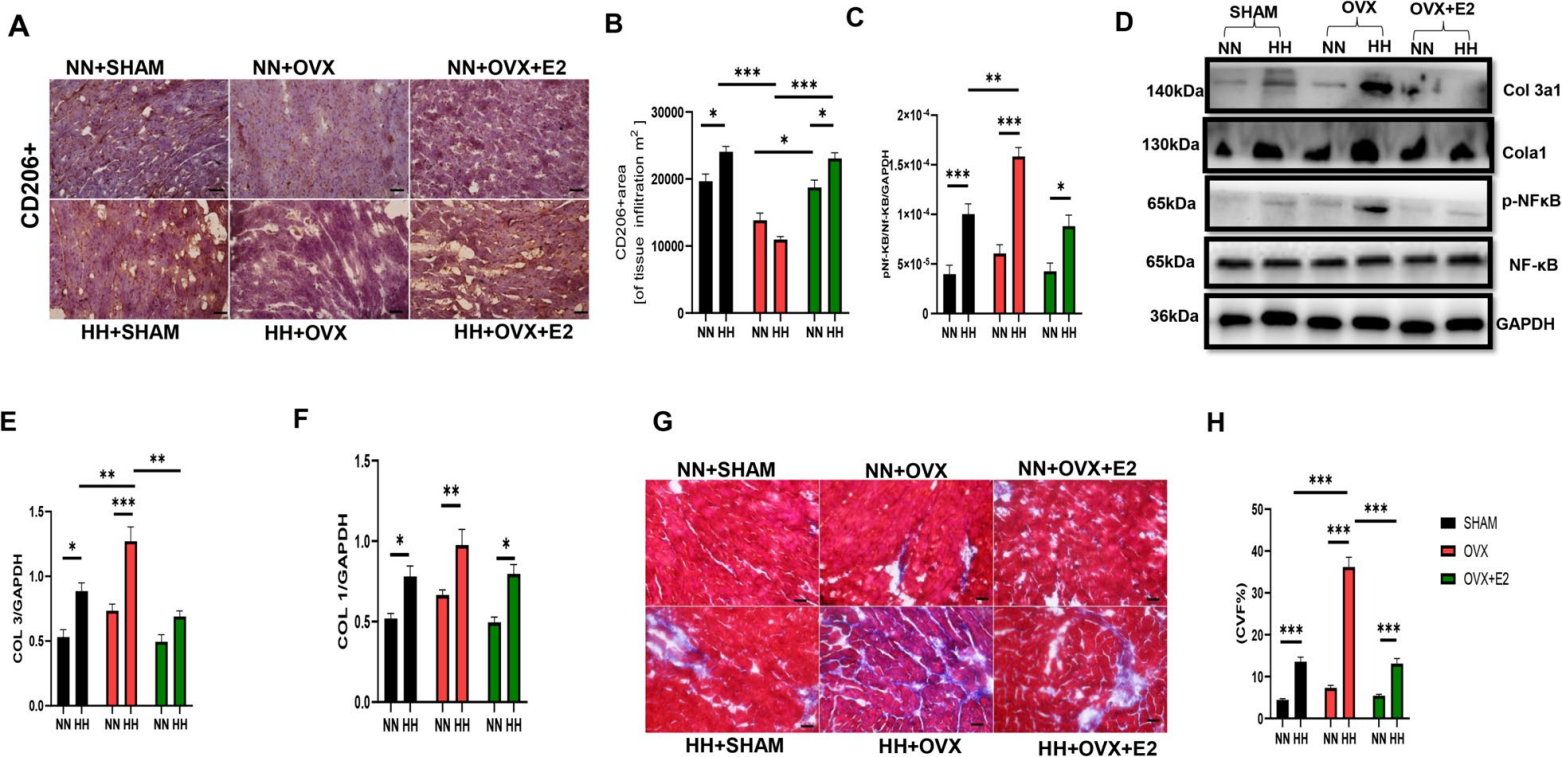
Effect of E2 on CD206+ Macrophage infiltration, NF-κB, and Fibrosis during HH. **A**-**B**: Representative Immunohistochemistry graphical presentation of CD206+. **C** Graphical Representation of NF-κB. **D**: Western Blot (**E** & **F**) Graphical Representation of Collagen Blot (**G** & **H**) Graphical representation of fibrosis and fibrotic staining of the myocardia. Results are presented as mean ± SEM. (n = 6) (*p < 0.05, **p < 0.01, ***p < 0.001)

Furthermore, our examination of macrophage infiltration in the myocardium revealed increased total macrophage (F4/80 +) infiltrations in the OVX + HH group (Fig. 4F and G), concomitant with increased infiltration of proinflammatory M1-like (CD86 +) macrophages (Fig. 4H and I). However, the presence of estrogen in the SHAM + HH and OVX + E2 + HH groups regulated macrophage polarization, favoring a shift from the proinflammatory M1-like (CD86 +) phenotype towards an anti-inflammatory M2-like (CD206 +) state, evidenced by the increased prevalence of CD206 + macrophages in these estrogen-replete groups compared to the estrogen-deficient OVX + HH cohort (Fig. 5A and B). We observed increased phosphorylation of the nuclear factor-kappa B (NF-κB) pathway, a key regulator of inflammatory responses, in the OVX + HH group relative to the estrogen-treated groups (Fig. 5C and D).

Assessment of cardiac fibrosis through Masson's trichrome staining revealed an association between HH exposure and increased fibrotic deposition compared to normoxic controls (NN). Moreover, estrogen deficiency exacerbated this fibrotic response, as the OVX + HH group exhibited a more severe fibrotic state than the SHAM + HH and OVX + E2 + HH groups (Fig. 5D–H). Corroborating these findings, collagen deposition within the myocardium was significantly elevated in the OVX + HH cohort, while the presence of endogenous (SHAM + HH) or exogenous (OVX + E2 + HH) estrogen mitigated collagen accumulation (Fig. 5G and H). These results depict hypobaric hypoxia as a condition associated with heightened inflammation and fibrosis in cardiac tissue, with estrogen deficiency potentiating these maladaptive responses. Conversely, estrogen appears to confer cardioprotective effects by suppressing excessive inflammation, modulating macrophage polarization, and attenuating fibrotic remodeling.

### GPER regulated the expression of MIF by optimizing HIF-1α expression during HH

**Fig. 6:**
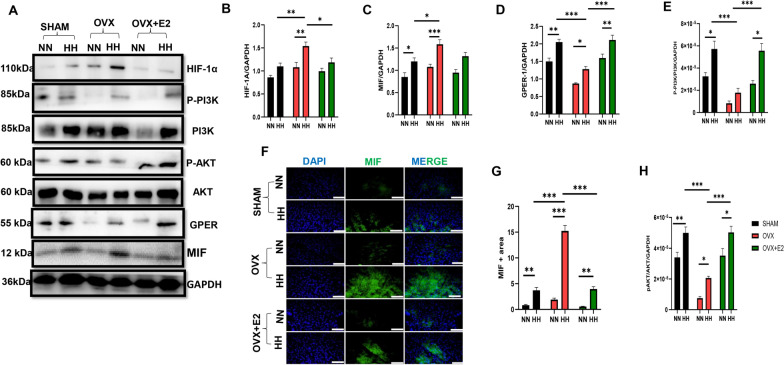
Effect of E2 on HIF-1a, MIF activity, and GPER pathway expression during HH. **A** Western blot band: **B**-**E** & **H** Representative immunoblots graphical presentations of HIF-1α, MIF, GPER pathway. **F** & **G** Immunofluorescence graphical representation. Results are presented as mean ± SEM. (*p < 0.05, **p < 0.01, ***p < 0.001), (n = 6)

**Fig. 7:**
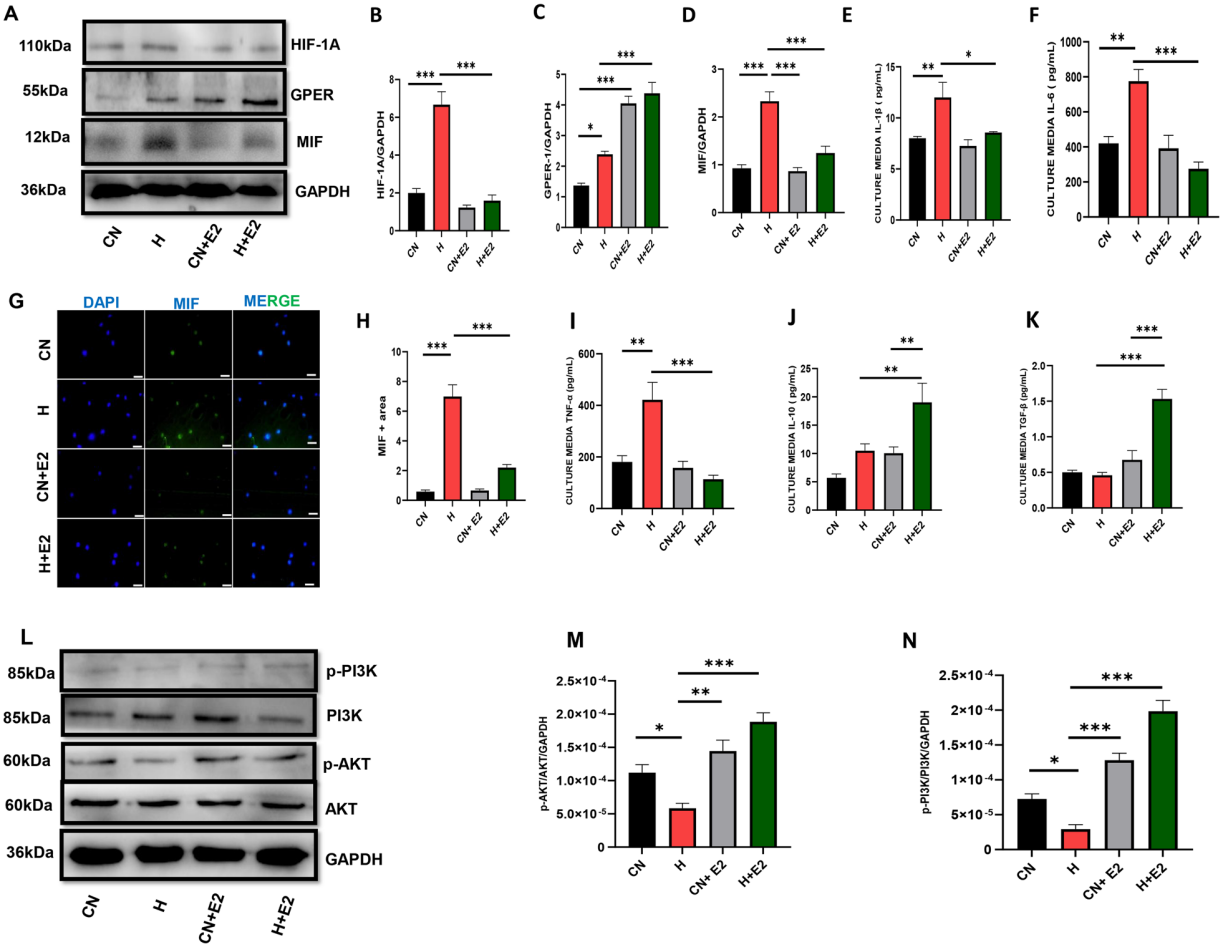
Estradiol (E2) downregulated HIF-1A and MIF inflammatory markers during HH in isolated macrophages. **A** Western blot bands **B**-**D** Graphical Representation of Western blot. **G** & **H** Immunofluorescence representation (**E**-**F**, **I**-**K**) Inflammation markers. **L** Western blot bands (**M**, **N**) Graphical Representation of blot. Results are presented as mean ± SEM. (*p < 0.05, **p < 0.01, ***p < 0.001), ONE WAY ANOVA

**Fig. 8:**
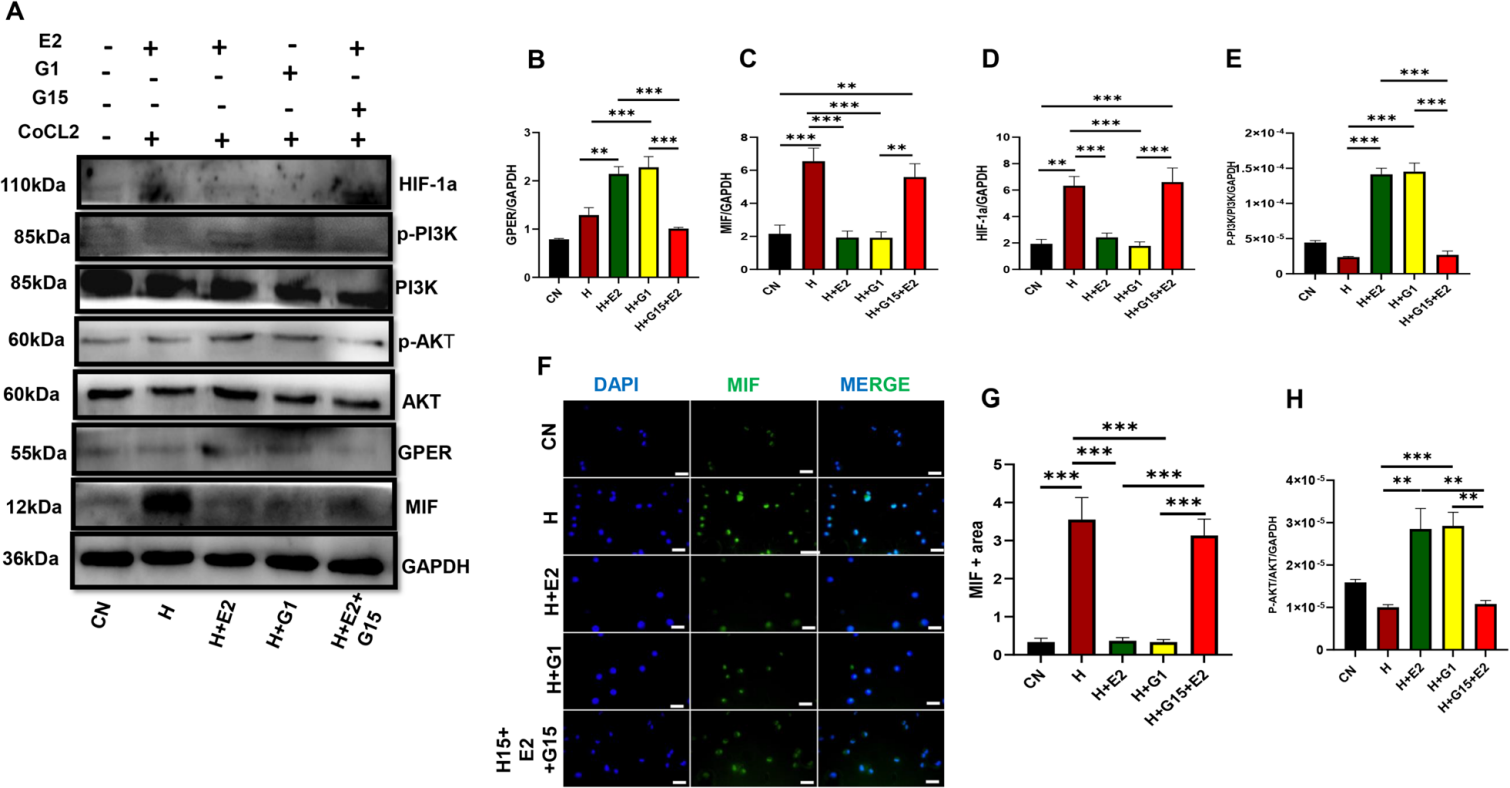
Effect of GPER on HIF-1α and MIF expression during HH: **A** Western blot bands (**B**-**E** & **H**) Graphical Representation of Western blot. **F** & **G** Immunofluorescence graphical representation. Results are presented as mean ±SEM. (*p<0.05,**p<0.01, ***p<0.001), ONE WAY ANOVA

During HH, the expression of HIF-1α, an indicator of the hypoxic state, and MIF were examined. The absence of estrogen was observed to upregulate the expression of these proteins (Fig. 6A−C, F and G). However, the estrogen downregulated its expression in the SHAM and OVX + E2 group during HH (Fig. 6A–C, F and G). Consequently, the expression of GPER, PI3K/AKT was upregulated in the presence of estrogen compared to the absence of estrogen during HH (Fig. 6A, D, E, H). Furthermore, in our in vitro study, we first confirmed the hypoxic state in the cell by checking for the expression of HIF-1α. We observed an upregulation of HIF-1α and further an upregulated expression of MIF which was attenuated with E2 treatment, likewise the inflammation state in the cell (Fig. 7A–K). Also, we observed an upregulated expression of GPER, PI3K/AKT during E2 treatment of the cell (7L–N). To confirm the pathway utilized by E2 in regulating HIF-1α and MIF, we blocked the GPER (G15 antagonist) and activated it by G1 agonist to observe its effect on the HIF-1α and MIF expression; our results show that the inhibition of these proteins upregulated the expression HIF-1α and MIF in the presence of hypobaric hypoxia in the HH + E2 + G15 group compared to the E2 + HH and HH + G1 treatment group (Fig. 8A–H). These results suggest that estrogen through the GPER pathway regulates MIF expression during HH.

## Discussion

The increase in ascent to higher altitudes initiates physiological responses, primarily hyperventilation and elevated heart rate, to counteract the effects of hypobaric hypoxia experienced by individuals at high altitudes. (Mallet et al. 2021) Acute exposure to hypobaric hypoxia induces physiological adaptations, including increased red blood cells (RBCs), enhanced oxygen utilization, increased pulmonary ventilation to compensate for the low oxygen state, and increased cardiac output to meet the body's demands. (Theunissen et al. 2022) However, prolonged stay at high altitudes and underlying conditions in individuals may lead to pathological outcomes upon exposure to hypobaric hypoxia. Chronic hypobaric hypoxia initiates maladaptive responses such as inflammation, metabolic shifts, and increased reactive oxygen species (ROS) activity (Pena et al. 2020). Studies have reported hypobaric hypoxia to be associated with arrhythmia (Adzika et al. 2023), and chronic hypobaric hypoxia was noted to initiate pathological responses, including pathological cardiac hypertrophy, inflammation, and metabolic shifts. (Nishimura et al. 2023).

The ascent to high altitude places a compensatory demand on the heart, increasing cardiac output and sympathetic activity (Ebihara et al. 2023). A prolonged stay increases the risk of cardiac arrhythmia by increasing heart rate and sympathetic overactivity (Mallet et al. 2021). Our study consistently reported prolonged QT interval, QTc heart rate, JT interval, and ST height, which are arrhythmia indicators, in ovariectomized mice kept in HH, corroborating the occurrence of arrhythmia during HH while underscoring the critical role of estrogen deficiency in cardiac outcomes during HH, compared to those kept in normoxia (NN) and estrogen-treated mice. The control mice at NN had improved cardiac electrical activity compared to those in HH conditions. Estrogen, a known regulator of heart function, attenuates excessive sympathetic activity and enhances parasympathetic tone to improve heart function. Our ovariectomized estrogen treatment groups experienced a mitigated heart rate and a shortened QT, QTc, ST height, and JT interval, thereby lessening the likelihood of arrhythmia compared to the estrogen-deficient group. The presence and absence of estrogen highlight the importance of hormonal balance in maintaining cardiac health under hypoxic conditions. (Verratti et al. 2017; Savji et al. 2018).

Prolonged high-altitude exposure may cause structural changes to the heart including ventricular hypertrophy. Hypobaric hypoxia triggers compensatory hypertrophy and an increase in the induction hypertrophy mediator gene in regulating the low oxygen state (Pena et al. 2020). GATA 4 mediates hypertrophy-related genes such as BNP and ANP which also stand as cardiac injury markers (Kohli et al. 2011). However, its sustained activation exacerbates cardiac hypertrophy (Rysä et al. 2010). During HH, the ovariectomized hypobaric hypoxia group experienced increased cardiac hypertrophy, as evidenced by increased body weight, BW/HW ratio, and cardiomyocyte area compared to the estrogen treatment group. Furthermore, our study observed an increase in right ventricular hypertrophy in the OVX + HH group, which was alleviated by estrogen treatment. This is consistent with the other studies that observed an increase in RV hypertrophy compared to the LV hypertrophy (Zhang et al. 2022). The absence of estrogen makes females prone to increased cardiac mass, leading to cardiac hypertrophy. GATA4 is a transcription factor that mediates cardiac hypertrophy and is a known regulator of hypertrophic-related genes such as BNP and ANP in the heart. (Afouda 2022) The expression of GATA 4 was upregulated during hypobaric hypoxia in the ovariectomized group. In contrast, the presence of estrogen downregulated its expression, in the Sham and estrogen-treated hypoxia groups. Although GATA 4 is known to mediate cardiac hypertrophy, its expression was pathological in estrogen-deficient mice under HH compared to NN condition depicting an exacerbated cardiac remodeling. However, the presence of estrogen downregulated its pathological expression, as estrogen is known to function as an anti-hypertrophy mediator. (Haines et al. 2012) Furthermore, hypertrophy–related genes such as BNP and ANP in the ovariectomized mice under HH were increased compared to the Sham and estrogen-treated HH group. The increase in BNP, ANP, and cTnI in the ovariectomized group depicts myocardial hypertrophy. Estrogen's cardioprotective function relates to the modulation of pathological cardiac hypertrophy (Haines et al. 2012). This was observed in its regulation of body/heart weight, GATA 4, ANP, BNP, and cTnI expression, indicating the therapeutic role of estrogen in combating hypertrophy and improving the heart's structure at a low oxygen state.

Inflammation is a characteristic feature of maladaptive response during high-altitude acclimatization (Pham et al. 2022). Such phenomena become excessive under prolonged exposure to hypobaric hypoxia. The stabilization and persistent signaling of HIF-1α upregulates inflammation-related genes and the expression of pro-inflammatory cytokines such as MIF, IL-1β, TNF-α, and IL-6. (McGettrick and O’Neill 2020) Our results depicted a similar phenomenon with aggravated inflammation with the upregulated expression of MIF, IL-1β, TNF-α, and IL-6. This inflammation was regulated by estrogen, as the SHAM and OVX + E2 + HH groups had downregulated expression of HIF-1α and MIF, decreased IL-1β, IL-6, and TNF-α levels, and increased levels of anti-inflammatory cytokines such as IL-10 and TGF-β. Transforming growth factor-β (TGF-β) is a key regulator of tissue remodeling and extracellular matrix (ECM) deposition. Its excessive signaling leads to fibrosis, characterized by excessive collagen deposition and tissue stiffening in pathological conditions. (Verrecchia and Mauviel 2007) However, it also functions as an anti-inflammatory cytokine. It inhibits NF-κB activation, reducing the production of pro-inflammatory cytokines such as TNF-α, IL-1β, and IL-6, and also modulates immune cells which facilitate inflammation. (Sanjabi et al. 2017) Our work observed a concurrent reduction of IL10 and TGF-β mirrored in an increase in proinflammatory cytokines indicating that TGF-β may be playing an anti-inflammatory role. This is evidenced in an increase in collagen deposition despite the reduced TGF-β levels in the ovariectomized group. Also, the decreased IL-10 and TGF-β levels corresponded with increased NF-κB activation and p-NF-κB levels. Furthermore, estrogen treatment groups experienced an increased IL-10 and TGF-β while decreasing NF-κB, collagen deposition, and pro-inflammatory cytokines. So, the presence of estrogen promoted TGF-β increase which might promote the anti-inflammatory effects of TGF-β. (Hou et al. 2021) Estrogen has been shown to downregulate inflammation-related genes and pro-inflammatory cytokines during stress, leading to a decrease in inflammatory cytokines and an increase in anti-inflammatory cytokines (Ndzie Noah et al. 2023a), consistent with our findings. Additionally, there was a downregulated expression of Nf-κB, a known regulator of inflammation. Also, the hypoxic state attracts the infiltration of immune cells including macrophages which amplify the inflammatory response (Castillo-Rodríguez et al. 2022). Accordingly, we observed increased infiltration of total macrophages in the OVX + HH state, with a surge in CD86 + macrophage infiltration. Subsequently, CD206 + macrophage infiltration decreased, whereas, in the estrogen groups, there was an increased polarization towards CD206 + macrophages compared to CD86 + , along with a downregulation in MIF expression. Our results showed that the absence of estrogen exposes females to an exacerbated inflammatory state upon exposure to hypobaric hypoxia (HH). This condition might be possible in postmenopausal women and men, who experience reduced estrogen levels.

Hypoxia exacerbates cell damage by altering cardiac tissue in the presence of injury, potentially modifying the extracellular matrix and promoting fibrosis (Gilkes et al. 2014; Chen et al. 2020). In the liver, hypoxia has been shown to exacerbate fibrosis through HIF-1α-induced activation of fibrosis mediators and markers (Watson et al. 2014; Roth and Copple 2015; Triantafyllou et al. 2019). In chronic hypobaric hypoxia, the fibrotic state might be exacerbated. (Xie et al. 2022) Our results showed a worse state of fibrosis with an upregulation in collagen 1 and 3 deposition in the heart. Further staining of cardiac tissue exhibited an exacerbated state of cardiac fibrosis in the OVX + HH group compared to the estrogen-treated group, which had reduced cardiac fibrosis. Estrogen deficiency in women subjects them to cardiac fibrosis during hypobaric hypoxia, affecting cardiac function (Zhang et al. 2022). This demonstrates a favorable therapeutic effect of estrogen treatment during HH.

Estrogen's cardioprotective effects in the cardiovascular system involve the induction of its genomic and non-genomic receptors, estrogen receptor α (ERα), estrogen receptor β (ERβ), and G protein-coupled estrogen receptor (GPER), which modulate gene transcription to enable cardiovascular homeostasis. (Aryan et al. 2020) During cutaneous wound healing, estrogen has been shown to downregulate MIF expression via the ERα/PI3K pathway. (Gilliver et al. 2010; Horng et al. 2017) However, the role of the GPER pathway in regulating MIF in the heart during hypobaric hypoxia remains unexplored. Our study, to the best of our knowledge, provides the first evidence of this effect. We observed an upregulated expression of GPER during HH, which downregulated MIF and HIF-1α expression. HIF-1α stabilization under hypoxia allows the transcription of genes and processes to modulate physiological adaptation during HH, however, its sustained signaling becomes detrimental as it worsens inflammation, fibrosis, and cardiac hypertrophy (Lee et al. 2020; Sato and Takeda 2023). Therefore, the regulation of HIF-1α and MIF by estrogen through GPER may regulate its expression at an optimal level to curb any form of adverse response. This was more apparent in the estrogen-treated mice group than in the OVX group. Similar observations were made in the upregulated expression of PI3K/AKT in the presence of estrogen. This receptor was further examined in an in vitro study, where macrophages were utilized coherently with inflammation and the expression of MIF. We detected an upregulation in the GPER/PI3K/AKT pathway and a downregulation of MIF and HIF-1α. Furthermore, we administrated G15 (GPER antagonist) and G1 (GPER agonist) to peritoneal macrophages with CoCL_2_ to induce hypoxia and it revealed that GPER inhibition upregulated MIF and HIF-1α expression, whereas GPER activation by G1 downregulated their expression. Although the PI3K/AKT pathway was not further inhibited to ascertain the mechanism, previous research has shown that G15 or G1 administration affects the phosphorylation of the PI3K/AKT pathway (Adu-Amankwaah et al. 2023; Ndzie Noah et al. 2023b), which we also observed. HIF-1α can be detrimental during hypoxia by inducing apoptosis in embryonic stem cells, leading to cell death. (Zhang et al. 2018) Our results showed upregulated PI3K/AKT expression alongside decreased HIF-1α expression. We consider this effect as being driven by estrogen as the upstream inducer of the PI3K pathway, rather than the hypoxic state being the primary regulator of the pathway.

Furthermore, estrogen's regulatory effects on inflammation during hypobaric hypoxia extend beyond the cardiovascular system. Estrogen has been shown to modulate the immune response, reducing proinflammatory cytokines and increasing anti-inflammatory cytokines, thereby providing a protective effect against inflammation-induced damage in various tissues (Harding and Heaton 2022). This hormonal regulation is crucial in mitigating the adverse effects of prolonged hypoxia and maintaining overall physiological homeostasis.

The role of estrogen in cardiac protection is multifaceted, involving direct effects on cardiac cells, modulation of gene expression, and interaction with signaling pathways that regulate cell survival, growth, and function (Ueda et al. 2019; Knowlton and Lee 2012). Estrogen's ability to upregulate protective pathways and downregulate harmful ones underscores its potential as a therapeutic agent in conditions of chronic hypoxia and other stress-related cardiac conditions.

In summary, our findings emphasize estrogen's critical role in modulating both physiological and pathological responses to hypobaric hypoxia. Estrogen exerts cardioprotective effects by regulating cardiac hypertrophy, inflammation, macrophage infiltration, and fibrosis, highlighting its potential as a therapeutic agent to mitigate adverse cardiac outcomes during high-altitude exposure. The interplay between estrogen, its receptors, and various signaling pathways provides a coherent mechanism for its protective effects. Understanding these mechanisms could lead to targeted therapies that mimic or enhance estrogen's actions, offering new treatments for individuals exposed to high altitudes or those suffering from chronic hypoxia.

### Limitations and future directions

This study does not fully explore the downstream PI3K/AKT pathway, which could offer additional insights into estrogen's mechanisms. Future research should examine the effects of GPER modulation on MIF and HIF-1α gene expression during chronic HH exposure to validate and expand our findings.

## Conclusion

Our study underscores estrogen’s therapeutic potential in protecting against hypoxia-induced cardiac inflammation, fibrosis, and hypertrophy, with a mechanism involving GPER-dependent attenuation of HIF and MIF expression. These findings advance our understanding of maladaptive responses to hypobaric hypoxia and highlight the clinical potential of estrogen-based therapies in mitigating hypoxia-related cardiac complications.

## Data Availability

No datasets were generated or analysed during the current study.
