# Correction: Estrogen via GPER downregulated HIF-1a and MIF expression, attenuated cardiac arrhythmias, and myocardial inflammation during hypobaric hypoxia

**DOI:** 10.1186/s10020-025-01380-6

**Published:** 2025-11-12

**Authors:** Prosperl Ivette Wowui, Richard Mprah, Marie Louise Ndzie Noah, Joseph Adu-Amankwaah, Anastasia Wemaaatu Lamawura Kanoseh, Li Tao, Diana Chulu, Simon Kumah Yalley, Saffia Shaheen, Hong Sun

**Affiliations:** 1 Department of Physiology, School of Basic Medical Sciences, Xuzhou Medical University, 209 Tongshan Road, Xuzhou, 221004 Jiangsu China; 2 Xuzhou Key Laboratory of Physiological Function and Injury, Xuzhou Medical University, Xuzhou, China; 3 National Demonstration Center for Experimental Basic Medical Science Education, Xuzhou Medical University, Xuzhou, China

**Correction: Molecular Medicine 31, 107 (2025)**

**https://doi.org/10.1186/s10020-025-01144-2**

In this article (Wowui et al. 2025), Fig. 2 appeared incorrectly and have now been corrected in the original publication.

For completeness and transparency, the old incorrect versions are displayed below.

The original article has been corrected.

Incorrect Fig. 2.

**Fig. 2:**
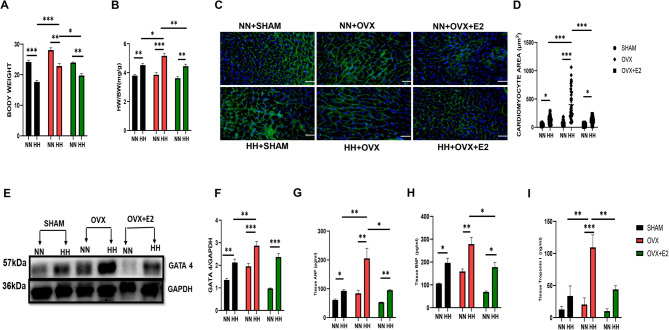
Effect of E2 on cardiac morphometric and injury markers. **A** & **B** Graphical representation of Body weight, and HW/BW. **C** & **D** Cardiomyocyte area. **E** & **F** Western blot band and graphical representation of blot **G**, **H**, **I**: Graphical representation of markers of hypertrophy and injury. This result is represented as a mean ± SEM. (*n* = 6) ( *p < 0.05, **p < 0.01, ***p < 0.001), ANP Atrial Natriuretic Peptide, BNP Brain Natriuretic Peptide, BW Body weight, cTnI Cardiac troponin I, ELISA Enzyme-linked immunosorbent assay, GATA4 GATA Binding Protein 4, HW Heart weight

Correct Fig 2.

**Fig. 2:**
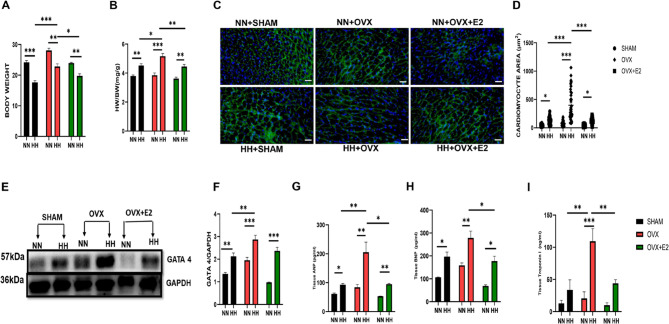
Effect of E2 on cardiac morphometric and injury markers. **A** & **B** Graphical representation of Body weight, and HW/BW. **C** & **D** Cardiomyocyte area. **E** & **F** Western blot band and graphical representation of blot **G**, **H**, **I**: Graphical representation of markers of hypertrophy and injury. This result is represented as a mean ± SEM. (*n* = 6) ( *p < 0.05, **p < 0.01, ***p < 0.001), ANP Atrial Natriuretic Peptide, BNP Brain Natriuretic Peptide, BW Body weight, cTnI Cardiac troponin I, ELISA Enzyme-linked immunosorbent assay, GATA4 GATA Binding Protein 4, HW Heart weight
